# Practical Hints Relating to Yellow Fever Prevention

**Published:** 1880-07

**Authors:** Robert B. S. Hargis

**Affiliations:** Pensacola, Florida


ARTICLE II.

### PRACTICAL HINTS RELATING TO YELLOW FEVER PREVENTION.

BY ROBERT B. S. HARGIS, M. D., PENSACOLA, FLORIDA.

The signs of the current season are such as I have known to precede Yellow Fever Epidemics. The history of the disease in this region is that there are years when the spread seems to be impossible and there are others when all efforts fail to stem its course. The condition essential to an out-break is the arrival of infected shipping; but from 1853 to 1861, I treated cases annually, in vessels in our bay, and, coming ashore, made no disguise of my mission, without exciting surprise, much less fear. The virulent epidemic of 1853 originated in New Orleans on the arrival of the ship Northampton, direct from Liverpool, with emigrants. False swearing failed to hide the deaths from yellow fever, which occurred in the Caribbean sea, or the cases presenting themselves in the gulf, and amongst newly landed passengers. I personally investigated this case and know that the vessel never touched land between Europe and New Orleans, and was protected from any other sources of contamination than that incidental to traversing the dangerous Atlantic calm belts under conditions favorable to yellow fever development. The out-break occurred early in the season, in May, and we became the victims of an extensive epidemic, which had been introduced from abroad by an infected vessel.

It was this case of the Northampton and its resultant evils, which made me waver in opinion as to the possible land origin of the disease and which modified the course of my subsequent investigations, observations and thoughts. The lesson was too striking to produce any but the strongest impression, and I soon familiarized myself with the contrasts exhibited by the numerous ships exhibiting signs of having been poisoned in infected ports, and of those in which the disease had evidently broken out spontaneously. The many opportunities for study in the eight years preceding the war, satisfied me, first, that the epidemic of 1853 had protected the majority of our people, hence the uncertainty of infection from communication with the shipping. Secondly, that the persistent gulf breeze which renders Pensacola one of the most delightful cities in the south for summer residence, in all probability exercises a marked protective influence against yellow fever, which revels in stagnant air. Thirdly, that the periods of high temperature are not necessarily the periods of greatest danger to human life; but cold nights succeeding hot days, seem to check the discharge from the system of poison which might otherwise be forced out, and develop many cases of an aggravated type.

Fourthly, that in years when an excess of ozone in the atmosphere in spring, is attended by almost epidemic, and very severe catarrhs, we are apt to have an autumn with an atmosphere deficient in ozone, well adapted to favor the spread of yellow fever. No example was more startling than in the condition of matters in 1878, when we escaped the epidemic by rigid quarantine. In no year have I noticed better defined indications, which personal experience causes me to reckon as of special importance.

We are having a repitition of 1878. I do not attempt to foresee the meteorological features of the succeeding months, but we have had the catarrhal fevers and now we must look out against disease importation. No one is more anxious than I am of the slight scientific value of these generalities, which are mentioned to direct inquiries and not to stifle investigation. As practitioners of medicine, we form personal opinions from local knowledge which help us materially in our work, and soon we shall have much greater precision and more rational deductions. I can, however, confirm an almost accepted truth in epidemiology, that the careful observer must watch the vital statistics of seasons preceding epidemics, to obtain useful etiological information. It is this which has inspired me with hope or fear when witnessing the first instance of yellow fever in the shipping in our port. In some cases I felt sure of exemption, and in others, of fatal consequences.

Western Florida has furnished me with some singularly definite experiences, and whilst at one time witnessing the malady on the cleanest, driest and healthiest regions, as at Milton and Fort Barrancas, I can testify to the virulence of the malady in unwholesome and over-crowded dens. Here we have, fortunately, in winter, a congregation of old hulks discarded for all other than lumber trade. The many annually lost and reported waterlogged on the broad ocean, were not liable to yellow fever. When the pumps have to be kept going all the time, it is difficult, but by no means impossible to have fever developed. These old and defective vessels have afforded me personal experience in summer of the poisonous character of these emanations, for it was by concentrated ship poison I caught yellow fever in 1853, after having past unscathed through a number of epidemics.

The malarial elements on the Escambia, distinctly limited now as it was in the days of the French Huguenots, cited by Lind, cannot travel in ships at sea ; agues cease and sailors improve every day after they leave port, but it is quite otherwise on vessels which have either caught or developed yellow fever.

OCEAN INFECTED SHIPS.

The resident in a gulf port having daily intercourse with mariners, learns that the suffering at sea in the tropics are beyond description. Hailing from any quarter of the globe, the oppressed crews refuse to go below, and lie about on deck, seeking some means of relief by the working of the ship, in the deathly stillness of the "calms." At times a man ventures below and the foul forecastle seals his fate. He is buried at sea, having died of a nameless disease, which I know from analogous cases reputed by intelligent captains and medical men, to be generally yellow fever. But such a ship may enter our port, in ballast, without a death, and only an unusual sallowness and sickliness of the crew. They go ashore and arrange for discharging their ships and cutting ports to load lumber. The laborer and carpenters engage in these operations, are the first to succumb. They are specially susceptible and are often the first and only ones to reach the foul bilge. It is only those who have smelt the combined sulphuretted hydrogen and fetid ammoniacal odors who can appreciate the character of this poisonous gas, incapable of supporting combustion and animal life. Dead rats and roaches by the bushel are not rarities, and I have seen the active bubbling of gas on the surface of the bilge, supplying a constant quantity of putrid vapor, which effectually displaces or precludes the entrance of fresh air.

Ocean infected ships usually have rotten timbers, are not very leaky, come in sometimes from Rio with rock ballast, whereas, at others, they have a more dangerous ballast in the shape of coal, which is loaded in Wales to avoid the charge for relieving a vessel of other ballast.

The merchant ships of the future should have water ballast in perfectly tight tanks. Every part of the timber should be made impervious to wet and elements of organic decay.

A ship may have an infected hold as the result of infection from land, so that whilst specifying the distinctive features of the condition of a sickly ship entering our ports, after having left any European port without possible yellow fever infection, there are cases which may be confounded with it. Above all, we must state our positions fairly and truthfully, not attempting to prove too much, or reckless by disregarding sources of recorded facts after the manner of recent writers who declare the spontaneous origin of yellow fever in ships impossible.

SIMPLE FEVER CARRIERS.

I have vividly before my eyes the "Fair Wind," a vessel now in this port, and which is not a yellow fever ship like those in which the disease has originated *de novo*. She came here in July, 1867, as clean, as pure and well equipped as any vessel I have ever seen of her kind. She brought the yellow fever, and how? Amongst her crew, who contracted the disease at Kingston, Jamaica, before sailing for the port of Pensacola. After the discontinuance of the disease on board, and though disinfected in the uppermost deck, cabin, forecastle, &c., she opened her hatches and port holes and proceeded to load; not a single case occurred amongst the steamer's employees that could be traced to that source; the Fair Wind was a carrier but not a generator of the malady,

Land infected vessels show signs of the disease usually after leaving an infected port, and rarely beyond the 4th or 5th day. When a ship has been ten days at sea or over, the chances are she has a foul bilge and will infect all the susceptible people who enter her.

Vessels from Rio coming to this port, if infected in Rio, show signs of the disease unmistakably south of the equator and usually south of the tropic of Capricorn. If the out-break be delayed until the line is crossed, the permanent infection of the vessel originating in the ship is highly probable. Nothing but familiarity with this subject and with shipping can enable a sanitarian to appreciate the sharpness of these distinctions. The most puzzling complications are due to the vast difference between men as to constitutional pre-disposition. In yellow fever as in every disease, the study of diathesis, susceptibility, cause of immunity, may be placed in the first rank with a view to intelligent records.

Medical men especially connected with southern ports, should, in tracing the history of a ship, obtain from the log an account of her course. A sailing vessel from Europe making for New Orleans, has to keep far out on the Atlantic and strike a southerly course, to avoid the opposing gulf stream, and this is a circumstance which has specially attracted Mr. Gamgee's attention, accounting for the rarer out-breaks on vessels leaving for Europe, than those coming from Europe or sailing into the gulf from the southern hemisphere. Every degree north that is past affords a relative protection, but the winds and currents which impel the vessels northward, exert a favorable influence on the health of the crew and passengers of ships at sea.

Indeed, it is under these circumstances that even susceptible crews suffer little or not at all, in an infected ship, until she reaches a port and is opened out as in the celebrated case of the Anne Marie, which left Nantes for Havana on the 12th of May, 1861, started healthy on her return voyage on the 13th of June, and seventeen days after leaving port had a case of yellow fever on board. The disastrous consequences which ensued in and around St. Nazaire, in the north of France indicates that the northward travel of yellow fever is not impossible though ships are much relieved by stiff trade winds.

THE PERIOD OF INCUBATION OF YELLOW FEVER.

Slowly but surely have my eyes been opened to the folly of confounding natural phenomena in relation to disease. I appeal to medical men to abandon the talk about "germs" until "germs" are proved to exist. I have sinned like my colleagues, but the evils attendant on the use of words which do not implicitly convey a truth, has taught me a lesson. It is a fact that the development of fecundated ova, the embryonic stage of every known organic form, demand specific and invariable periods of time. A female acarus lodging on the human skin, has to produce the germs to produce the general and appreciable signs of itch. This takes time. The poison of small pox requires a definite and almost invariable period of latency or incubation, to overwhelm a healthy person. Not so with yellow fever. All the statements made in the past must be carefully scanned and it will be found that where a period of latency exists between exposure to the poison and manifestation of its effects, this period is usually very brief and extremely uncertain. It varies with the cases and with the epidemics. The simultaneous manifestations of the disease in two or more persons exposed at the same time to the poison, often observed, may be explained on the principle of poison doses. There are many drugs given in like quantities to two similar individuals, at the same time which produce a definite result at the exact same time. But strengthen the dose, and as with yellow fever, the effect is immediate. From the earliest careful observations of this disease, produced by foul ships, on susceptible people in ports, the instant sickness of persons who have breathed the air of the hold, has been noticed. I returned to shore from an infected ship in 1853, and remarked to friends who met me, that I was never so sick before in my life as I was then. They gave me brandy; I rejected it and took another drink with like effect. All the premonitory signs of fever seized me at once, and next morning I was transferred to Milton, which I reached in an unconscious state, and remained so more or less, for 6 days, with marked symptoms of yellow fever. I convalesced on the 7th day from the time of exposure and commencement of sickness. The same season a carpenter who left Milton as a refugee to escape yellow fever, went to Coffee county, Alabama, and returned after the first frost. He went straight to the house of his late friend Trammel, who had died in the summer; the house had been kept warm and after entering Trammel's bed he was forthwith seized with the premonitory signs of yellow fever and I treated him.

These are not rare though somewhat exceptional cases, and the experience in northern ports that people who have remained healthy 5 or 6 days at sea, may be regarded as free from the poison, is to a great extent indicative of the prompt action of the yellow fever poison. The type of the disease varies within certain limits strikingly, but every time has the same uncertain invasion which is totally unlike the latency and invasion of true fever. The well marked supre-orbital and other pains, with or without rigors, supervene in one, two, three or four days and rarely longer.

These points have a most important bearing on the prevention of the disease and guided by them, we can readily understand how ship infection, the inevitable precursor to port infection, must be controlled, whilst the people carried are much more easily dealt with, owing to the brevity of the latent stage. In six years we have had no yellow fever, but quarantine measures have been fairly carried out and have sufficed in conjunction with natural tendency to exceptional atmospheric purity, thanks to persistent natural winds peculiar to this region of country.

Pensacola is by no means a model of sanitary arrangements. We might easily have efficient drainage, which we lack. We have plenty of space and porous soil, so that until the town is more populous and crowded, we may escape the visitations of typhoid and other fevers or great mortality from cholera, should it be introduced. We have probably the healthiest city in the United States and the infant mortality is exceedingly slight, I may define it a rural port sheltered by healthy piney woods, and thanks to the breeze free from the heat diseases of inland, and other cities on this and even more northern latitudes.

Well have I understood as a resident in Pensacola the remarks frequently printed on the salubrity of the West Indian Islands, such as Curacoa, and how exactly similar the history of yellow fever here is with that of any other tropical port.

### PREVENTION OF YELLOW FEVER.

Every eye witness of a city, during an epidemic of yellow fever, has occasion to deplore the absolute helplessness of citizens and physicians in controlling the spread or anticipating the course of the plague. The well founded opinions of the rarity of personal contagion, under certain circumstances, coupled with a large body of protected people as in New Orleans, may be recognized as having materially favored the congregation of gossiping citizens in infested houses and localities, and thus, even by the well, the disease has been carried into many a house. The time was when, with less sanitary rule than is now enforced, a crop of yellow fever cases, led the curious and the charitable, the friends and the strangers who had no reason to fear an attack, to congregate, especially at the beginning of an outbreak, just like people run to a fire.

The now acknowleged transmissibility of the disease leads to the imposition of wholesome restrictions. Nevertheless taking a case like that of Memphis, depopulation and isolation failed to arrest the malady before frost. The reason for this is, in all probability, that the impure air, poisoned in almost unlimited masses, in hospital wards and houses, is free to move and passes laden from block to block, and street to street, until the sparsely inhabited suburbs and open country cut it short by abundant atmospheric dilution.

As a ship fever this malady is ever engendered by volatile matter, or matter suspended in moist warm air, emerging when the hold is opened, to poison the forecastle, the cabins, adjoining ships and houses. A slight breeze hastens its transit but often neutralizes it. It does not go far. A contaminated house on a wharf does not transmit by fomites, usually carried by man, the disease to a distant point of the port. It's proximate growth and a continuous current are marked in its course and stages by human victims.

I quite concur with Mr. Gamgee, that currents of pure air driven through and through infected places, constitute the most reliable means to dilute and ultimately to destroy the poison. The only danger is, that atmospheric dilution up to the present time has implied a city's doom—a wholesale infection. In grappling with the great difficulties which present themselves, as a believer in the oceanic character of yellow fever, I must first and foremost attend to ship sanitation. Medical men are not engineers and ship builders. Had they known how to advise, the practices commonly witnessed in gulf ports would long since have ceased.

The problems relating to ship ventilation and pumping the bilge water are of the highest importance, but I could not treat these subjects technically and I desire to restrict my observations to a few reforms and procedures which admit of prompt attention.

So far as the ship is concerned it should not be emptied of solid cargo until it is freely exhausted of its gaseous contents. All that is then removed from the ship should be freely aereated by positive currents of air, and in many cases there is no objection to sulphurous acid fumigation. The empty ship can then receive in its bilge some fresh burnt lime, and this may be sprinkled with advantage where foul gases may emanate. The word cleanliness imputes then, all that has to be secured; and by cleanliness and free ventilation, with thorough liming, a vessel can be purified; if, however, a low temperature can be secured, any way below 32° Fahr. and made to penetrate somewhat, the poison can certainly be destroyed.

M. Gamgee has suggested to me a means whereby the foulest timber can be purified by hot drying oils, which destroy all moist organic matter, and render the wood ever after impervious to moisture or elements of decay. The hot varnish could be applied to the whole interior of the ship with great advantage. An impermeable purifying coating may prove a great boon, and this constitutes one of the points which Professor Gamgee intends to carry out in his fever-proof ship.

The great advantage of having ships carrying skilled men and appliances for disinfection, is that infected vessels need never touch land until they are made safe. It is a bright idea and could undoubtedly be carried out in practice in our gulf ports.

Stations, such as the one at Ship Island, may be adopted, but we are threatened with that great impediment to common sense legislation in sanitary matters by divided counsels within states, and in adequate national administration. A sanitation could easily be provided for Havana by the Cuban government, and seeing how effectually the Spaniards have excluded yellow fever from their European Atlantic ports, it is to be hoped they may disregard the recently uttered statements that Cuba is to be the permanent hot bed of a naturalized pestilence.

I shall not attempt much on this occasion in relation to prevention of yellow fever on land, but I am happy to have been the first to witness M. Gamgee's experiments, whereby forced ventilation of rooms, with filtration and effectual disinfecting of air is rendered possible. He has designed an apparatus which he calls the "circlair," which enables us to shut up a room and practically isolate the volume of infected air which is abundantly purified and displaced by fresh air.

The poison which seems to travel on the roads and sidewalks, with the volume of heavy air dropping from infected houses, can in my opinion, be better destroyed by hot lime than carbolic acid. I object to this offensive compound, and believe that irritating and fetid vapors have only found favor because people have been led to believe they must do good, and that their presence was unmistakable.

I do not believe the yellow fever poison is difficult to destroy, but we must not be idle when a case indicates its location. Simple means will effect much, and I am in favor of prime attention to the foul gases of ships, the foul air of cities and notably of the sick room. Control these and much has been done to control the malady.

